# Observations on Mastodynia, or Milk Abscess

**Published:** 1821-08

**Authors:** John Burne

**Affiliations:** President of the Royal Medical Society, Edinburgh; and Member of the Royal College of Surgeons, London.


*Observations on Mastodynia, or Milk Abscess.* By JOHN BURNE, President of the Royal Medical Society, Edinburgh; and Member of the Royal College of Surgeons, London.

IT is a custom too general amongst medical men to publish only such cases as have something novel or extraordinary to recommend them. Extraordinary cases at once command attention, they are perused with eagerness, and treasured up with care; whilst those of daily occurrence are negligently read, or altogether passed over. The human mind is naturally fond of novelty: I acknowledge it is interesting, but it is not in proportion useful. This is a point generally admitted, and it would be well if it were as generally acted up to.

The disease which forms the subject of the following observations is, as all know, of lamentably frequent occurrence; and, on this account, may, by some perhaps, be considered too common-place to be obtruded on the attention of the profession. But, whoever has witnessed the suffering and inconvenience which every female afflicted with it experiences and labours under,—has seen it continue for months, in spite of the surgeon's utmost skill,—has noticed the everlasting deformity which, in the most beautiful part of woman, it occasions,—has observed that, while it deprives the infant of one source of its nourishment, it renders the other less abundant and less wholesome,—and has remarked that it deranges the health and impairs the strength of the anxious mother, at a time when both are most needed to nurse and cherish her infant;—he, I am satisfied, will agree with me, that milk abscess merits the especial consideration of every member of the profession.

Females are subject to milk abscess during the whole period of suckling, but they are more especially liable to be affected with it immediately after delivery; and it is to the abscess which is wont to occur at this particular time that my remarks will be confined.

The character of this abscess is phlegmonous, more or less, and its formation in the breast is attended by the same phenomena as the formation of phlegmonous abscess in any other part of the body. On the second or third day after delivery, when a copious secretion of milk has begun, the attack commences, and is marked by fullness, general tension with more or less hardness, and an uneasy sense of weight in one or other of the breasts: these symptoms are shortly succeeded by tumor, heat, redness, pain in some part, very commonly the upper and outer, of the breast affected, together with symptomatic fever. Slight shiverings now quickly supervene, and the suppurative process is established. The abscess, if not interfered with by the officious surgeon, bursts spontaneously, usually between the ninth and fourteenth days; the matter then escapes, and all the symptoms, local and constitutional, are alleviated.

The treatment of milk abscess is very simple,—the less, indeed, is done the better. Emollient fomentations with scalded plant-leaves suffice for the inflammatory stage, and oatmeal or linseed poultice for the suppurative. The same kind of poultice should also be continued after the bursting of the abscess, and until it be perfectly healed, be the length of time what it may. Unguents and like dressings do not sufficiently encourage discharge, whence it happens that, where they are employed, inflammation and suppuration again take place, and these occasionally more than once; whereas, if the poultice be uninterruptedly continued, all does well.

During the inflammatory stage, many recommend the local abstraction of blood by leeches, and the use of discutient lotions: the former treatment I have ever seen superfluous, and the latter injurious; the abstraction of blood protracting the inflammatory, and not preventing the suppurative, stage; and the lotions often causing a revulsion, or, in other words, often driving the disease from a situation not dangerous, to one, perhaps, highly so. Nothing, indeed, can be more likely to superinduce mania, or any other of the frightful diseases which are apt to afflict childbed women. For this reason, the employment of repellants cannot be too severely reprobated and condemned.

The indisposition of milk abscess to heal may possibly be owing to the great determination to the breasts during the first months of suckling; and, indeed, it is not unreasonable to suppose that nature keeps this, as it were natural issue, open, to counterbalance the dangerous plethora that might otherwise happen from the suppressed secretion of milk.

Such is the course, and such the treatment, of milk abscess; but the point of the greatest moment, and to which I am anxious to draw the attention of the profession, is the prevention both of abscess and of inflammation.

The frequency of milk abscess in the human species, and the rareness of it in the brute creation, cannot fail to strike the observant mind. The just-yeaned lamb empties without difficulty the loaded dug of its parent ewe, and the fresh-dropped colt the distended udder of its dam; but the new-born infant, although healthful and strong, often strives in vain to draw milk from the gorged bosom of its mother. The attentive nurse applies it again and again, but without avail; the milk accumulates more and more, and inflammation and abscess succeed.

The cause of failure is not with the infant, for it sucks well: the impediment, therefore, must exist on the part of the mother, and examination of the breast immediately discovers this to be the case; for the nipple is invariably found to be so flat and imperfectly formed as not to afford hold for the child.

There is no reason in nature why the nipple in the human species should be so very often incompletely developed, while the teats of animals are, without exception, as far as I know, prominent and perfect. The only cause to which the defect can be attributed is the continued pressure which, from the peculiarity of the female dress, (and of one part of it in particular, the stays,) the bosom is subjected to from childhood; and it is this pressure, without doubt, which prevents the full development of the nipple.

The imperfect nipple is the exciting, and distention of the lactiferous ducts the proximate, cause of milk abscess: it is, therefore, manifest that, if a proper nipple can be formed so as to admit of the milk being abstracted, abscess will be prevented.

The formation of a good nipple is by no means difficult: so far, indeed, from this being the case, I believe that it is always practicable; and the means by which it can be accomplished is by the breast-pump.

In a few hours after delivery, it is incumbent on the accoucheur to examine the patient's breasts, to ascertain whether or not the nipples are sufficiently prominent; and, if one or both prove otherwise, he should immediately set about to remedy the defect, for, by the early and repeated use of the pump, the nipple may be well drawn out before the secretion of milk takes place.

The pump should be applied over the flat nipple, and the degree of exhaustion of the air from the glass should be regulated by the feelings of the patient: the nipple will protrude into the mouth of the glass, and may be kept there two or three minutes; the pump is then to be taken off, and in the space of two minutes more to be again applied; and in this manner five or six times successively; and, on its being finally removed, suction should instantly be performed by the nurse for several minutes, for, unless it be done instantly, the nipple retires. The application of the pump must be repeated every three or four hours, as long as it may be required; taking care always that the nurse or infant suck that breast immediately on the removal of it. In this way, the accoucheur will never fail to procure good nipples, and, by so doing, to preserve his patient from all the inconveniences and sufferings attendant on milk abscess.

It is true that all this gives the practitioner a great deal of trouble; but it is amply recompensed by securing to him the good wishes and interest of his patient, and by increasing his reputation.

Experience allows me to affirm that the above-described method will prove successful, provided it is practised with attention and perseverance; for I have myself succeeded where both nipples did not only not project, but were considerably depressed.

The pump is rarely had recourse to with any other view than to abstract milk from the breast; but to this end it is quite inadequate, which may be the cause why it is not in more general use. In using the pump, it is advantageous to anoint the fore-part of the breast with oil, for this very much facilitates the entrance of the nipple into the mouth of the glass, and preserves the skin from abrasion.

*Edinburgh; June 1st*, 1821.

